# *In vitro* mechanistic study on mycophenolate mofetil drug interactions: effect of prednisone, cyclosporine, and others

**DOI:** 10.3389/fphar.2024.1443794

**Published:** 2024-08-26

**Authors:** Junjun Mao, Feifei Yu, Weiwei Qin, Guixian She, Yi Rong, Zhuohan Hu, Mingkang Zhong

**Affiliations:** ^1^ Department of Pharmacy, Huashan Hospital, Fudan University, Shanghai, China; ^2^ Vigonvita Life Sciences Co. Ltd., Suzhou, China; ^3^ Research institute for liver diseases (Shanghai) Co. Ltd., Shanghai, China

**Keywords:** mycophenolate mofetil, cyclosporine, prednisolone, salvia miltiorrhiza, drug-drug interaction

## Abstract

**Objective:**

The metabolism- and transporter-based drug-drug interactions (DDIs) between mycophenolate mofetil (MMF) and co-administered medications may be key factors for the high individual variability in MMF exposure. This study systematically assessed the influence of co-medications on the mycophenolic acid (MPA) pharmacokinetic (PK) process *in vitro*, particularly to provide mechanistic evidence of the metabolic interaction among steroids, cyclosporine (CsA), and MMF.

**Methods:**

Based on a previous study, we hypothesized that there are three main DDI pathways affecting MMF PK *in vivo*. A human hepatocyte induction study, transporter substrate/inhibition study using human embryonic kidney 293 cells, and multidrug resistance-associated protein 2 (MRP2) substrate/inhibition study using vesicle membrane were conducted to assess the mechanistic evidence of the metabolic interaction in triple therapies. The potential DDI risks associated with seven medications commonly co-administered with MMF in clinical practice were further evaluated.

**Results:**

The *in vitro* results suggested that prednisolone, the active metabolite of prednisone, induces the enzymatic activity of uridine 5′-diphospho-glucuronosyltransferase (UGT), particularly the UGT1A9 and UGT2B7 isoforms, resulting in increased metabolism of MPA to MPA glucuronide (MPAG). This induction potential was not observed in CsA-treated human hepatocytes. CsA inhibits organic anion-transporting polypeptide (OATP) 1B1- and OATP1B3-mediated MPAG. Prednisolone and CsA showed no inhibitory effect on MRP2-mediated MPAG efflux. *Salvia miltiorrhiza* significantly inhibited organic anion-transporting polypeptide and OAT 3 activities, suggesting that it affects the hepatic uptake and renal excretion of MPAG, causing increased MPAG exposure *in vivo*.

**Conclusion:**

These identified factors may contribute to the high inter-individual variability in MMF exposure and facilitate further development of mechanistic MMF PK models and individualized therapies.

## 1 Introduction

Mycophenolate mofetil (MMF), an ester prodrug of mycophenolic acid (MPA), is the predominant antimetabolite immunosuppressant that is used in combination with tacrolimus (TAC) or cyclosporine (CsA), with or without corticosteroids to prevent allograft rejection following solid organ transplantation (Hart et al., 2019). A time-to-event model study indicated that MPA exposure predicted better composite efficacy end-points (including acute rejection episodes, graft loss, and death) than calcineurin inhibitors exposure in patients undergoing renal transplantation (Daher Abdi et al., 2014). However, MMF is characterized by high variability in its metabolism and pharmacokinetics (PKs), which may lead to drug toxicity or reduced efficacy (de Jonge and Kuypers, 2008). Therefore, personalizing the MMF dose regimens is crucial.

When orally administered, MMF is absorbed and rapidly hydrolyzed by carboxylesterase into MPA, which is its active component. MPA is primarily metabolized by uridine 5′-diphospho-glucuronosyltransferases (UGTs) to form the abundant but inactive 7-O-mycophenolic acid glucuronide (MPAG) and the relatively minor but active acyl-glucuronide MPA (Kiang and Ensom, 2016). Organic anion-transporting polypeptides (OATPs, gene symbol SLCO) actively transport MPAG from the circulation into hepatocytes (Picard et al., 2010). Subsequently, MPAG is excreted into the bile *via* multidrug resistance-associated protein 2 (MRP2, gene symbol ABCC2) and breast cancer resistance protein (BCRP, gene symbol ABCG2) (Naesens et al., 2006; Miura et al., 2008). Following biliary excretion, MPAG undergoes enterohepatic recirculation (EHC), a process that contributes to approximately 40% (range: 10%–60%) of the total MPA exposure (Bullingham et al., 1998) and results in a second plasma peak of MPA between 4 and 12 h after administration (Jiao et al., 2008). Finally, circulating MPAG is filtered and actively excreted by renal tubular cells, with approximately 87% of the absorbed MMF eliminated in the urine by MRP2 (van Schaik et al., 2009).

A 10-fold variation in MPA exposure has been observed even in patients who were administered a similar dose during the first 2 weeks after kidney transplantation. MPA exposure was 30%–50% lower in the early phase after transplantation than in the stable period when a similar dose of MMF was administered (Shaw et al., 2003). Extrinsic (e.g., drug-drug interactions [DDIs]) and intrinsic (e.g., physiological variables and genetic polymorphisms) factors may influence the MMF PK process and contribute to high variability (Rong et al., 2022).

CsA reduces MPAG excretion into the bile and subsequent reabsorption of MPA through EHC by inhibiting the expression of MRP2 and OATPs in hepatocytes (Benjanuwattra et al., 2020), thereby reducing MMF exposure by 40% by inhibiting EHC (van Gelder, 2021). However, TAC has no clinically relevant effect on MPA PKs (Pou et al., 2001).

The steroid-MPA PK interaction is also a concern in triple therapies. Especially, the significant reduction in MPA clearance as a function of post-transplantation time may be explained by the diminishing enzyme induction effects associated with tapering steroid dosages over time (Cattaneo et al., 2002; Staatz and Tett, 2007; Rong et al., 2019). Regarding the drug interaction potential, various preclinical human and animal *in vitro* models have demonstrated that corticosteroids can induce the expression of UGTs (Schuetz et al., 1986; Kuno et al., 2008), altering the expression of MRP2 (Qadri et al., 2009) and organic anion transporter 3 (OAT3) (Wang et al., 2018), or displacing MPA protein binding (Morassi et al., 2018). Despite multiple reviews suggesting that corticosteroids may induce MPA metabolism (Staatz and Tett, 2007; Sherwin et al., 2011; Bergan et al., 2021), definitive clinical evidence supporting a direct steroid-MPA PK interaction is lacking. Moreover, opinions on the steroid-MPA PK interaction are contradictory (Rong and Kiang, 2023).

Other commonly co-administered drugs, in addition to the DDI between triple immunotherapies, may also influence the MMF PK process. Xu et al. evaluated the metabolism- and transporter-based interactions between nine co-medicated injections and TAC and found that DDIs may be the major cause of high individual variability in TAC (Xu et al., 2021). We evaluated the influence of seven drugs on the MMF metabolic pathway. These drugs are usually co-administered with MMF in China and include cefoperazone, pantoprazole, meropenem, probenecid, alprostadil, ganciclovir, *Salvia miltiorrhiza*, and olaparib.

The metabolism- and transporter-based DDIs between MMF and co-administered medications may be key factors responsible for the high individual variability in MMF exposure in patients who have undergone solid organ transplantation. Furthermore, systematic *in vitro* evaluation of MMF with co-medications may help elucidate the exact molecular mechanisms of DDIs, which may facilitate the development of mechanistic PK models to promote individualized MMF therapy. Therefore, this study aimed to systematically assess the influence of co-medications on the MPA PK process *in vitro*, particularly to provide mechanistic evidence of the metabolic interaction among steroids, CsA, and MMF.

## 2 Materials and methods

### 2.1 Materials

MMF (Product No. 100923-202003), MPA (Product No. 100924-202004), prednisolone (Product No. 100153-201405), and prednisone (Product No. 100199-201503) were purchased from the China National Institutes for Food and Drug Control (NIFDC). MPAG (Product No. M831520) was purchased from Toronto Research Chemicals, Inc. (Toronto, Canada).

Cefoperazone (Product No. R36425), pantoprazole (Product No. 313481), meropenem (Product No. 9160629101), alprostadil (Product No. 3B096K), and ganciclovir (Product No. 160805-1) were purchased from Pfizer (New York, NY, USA), Takeda Bio (Tokyo, Japan), Haibin Pharmaceutical Company (Shenzhen, China), Tide Pharmaceutical Company (Beijing, China), and China Meheco Keyi Pharma Co., Ltd. (Wuhan, China), respectively. *Salvia miltiorrhiza* injection (Product No. 1603283) and olaparib (Product No. S50443) were purchased from Chiatai Qingchunbao Pharmaceutical Co., Ltd. (Hangzhou, China), and Shanghai Yuanye Biotechnology Co., Ltd. (Shanghai, China), respectively.

Omeprazole (Product No. PHR1059) and β-estradiol (Product No. E8875) and its metabolite estradiol-3-glucuronide (Product No. E1127) were purchased from Sigma-Aldrich (Burlington, MA, USA). Additionally, 4-trifluoromethyl-7-hydroxycoumarin (Product No. 36852) and its metabolite, 4-trifluoromethyl-7-hydroxycoumarin glucuronide (Product No. T792035), were obtained from Sigma-Aldrich and Shanghai ZZBIO Co., Ltd. (Shanghai, China), respectively. Cryopreserved primary human hepatocytes (Lot No. HVN), as well as plating and incubation media, were purchased from BioIVT (Westbury, NY, USA).

Recombinant human embryonic kidney (HEK) 293 cells expressing human OATP1B1 (HEK293-OATP1B1, Product No. GM1102G), OATP1B3 (HEK293-OATP1B3, Product No. GM1106G), OAT1 (HEK293- OAT1, Product No. GM1103G), OAT3 (HEK293-OAT3, Product No. GM1104G), and HEK293 cells transfected with an empty vector (HEK293-MOCK, Product No. GM1101G) were supplied by GenoMembrane (Tokyo, Japan). Estradiol-17β-D-glucuronide (Product No. E1127), p-aminohippuric acid (Product No. A1422), estrone-3-sulfate (Product No. E0251), probenecid (Product No. P8761), and rifampicin (Product No. R3501) were purchased from Sigma-Aldrich. Dulbecco’s modified eagle’s medium (DMEM, Product No. 11885-084), penicillin-streptomycin (Product No. 15140-122), and fetal bovine serum (FBS, Product No. 10099-141) were obtained from Gibco (Grand Island, NY, USA). The bicinchoninic acid (BCA) protein assay kit (Product No. 23225) was provided by Thermo Fisher Scientific (Waltham, MA, USA).

Vesicle membranes expressing human MRP2 (Product No. 0001) were supplied by GenoMembrane (Tokyo, Japan). The transporter kit (GM3010RD), including Reaction Buffer A2 (containing 50 mM of MOPS-Tris, 70 mM of KCl, and 7.5 mM of MgCl_2_), Stop/Wash Buffer B (400 mM of MOPS-Tris, and 700 mM of KCl), 10 mM of MgATP and MgAMP solution and 200 mM of glutathione, used in the vesicle studies was provided by Research Institute for Liver Diseases (China). Benzbromarone (Product No. B5774) was purchased from Sigma-Aldrich.

### 2.2 Human hepatocyte induction study

Cryopreserved human hepatocytes were recovered in a pre-warmed incubation medium, diluted to a viable cell density (∼0.7 × 10^6^ cells/mL) using a plating medium, followed by the addition of 100 μL hepatocytes into each well of a collagen I-coated 96-well plate. The cell monolayer was formed after an overnight incubation period at 37°C under 5%CO_2_. The plating medium was then replaced with an incubation medium for further culture. On the second day, an incubation medium containing different concentrations of either compound, the inducer omeprazole (final concentration of 50 μM), or vehicle (0.1% [v/v] dimethyl sulfoxide [DMSO]). The cells were incubated for 96 h and additives were changed every 24 h. At the end of the incubation period, the medium was replaced with a substrate for UGT and MPA. Following a further incubation of 15 or 30 min at 37°C under 5% CO_2_, 100 μL of the reaction medium was transferred to 400 μL of ice-cold methanol containing internal standard (IS) to terminate the reaction. Finally, the supernatant was collected after centrifugation at 4,000 rpm for 10 min and analyzed for glucuronide formation using liquid chromatography-mass spectrometry (LC-MS).

### 2.3 Transporter substrate study using HEK293 cells

Briefly, cells were removed from liquid nitrogen and recovered at 37°C in a water bath and diluted to 0.8 × 10^6^ cells/mL with pre-warmed DMEM medium containing 10% (v/v) FBS. After seeding in 96-well plates pre-coated with poly-L-lysine at a density of 100 μL/well, the cells were pre-incubated at 37°C and 5% CO_2_ for 3–4 h to form a monolayer. Subsequently, the medium was removed and replaced with a fresh DMEM culture medium containing 10% FBS and penicillin-streptomycin solution (100 U/mL and 0.1 mg/mL) and incubated overnight.

Stock solutions of the compounds, substrates, and inhibitors were prepared using DMSO and further diluted to a dosing solution using a transporter buffer (TB). Before the substrate assay, DMEM was aspirated, and the cells were washed twice with 100 μL of pre-warmed TB. During the second wash, the buffer remained in the wells and was pre-incubated at 37°C for 5 min. Subsequently, TB was removed and replaced with 50 μL of TB-containing compounds at different concentrations or probe substrates with or without inhibitors, and the cells were incubated at 37°C for 10 min. Notably, MOCK cells were included in the compound and substrate groups to calculate the uptake ratio (UR) and its change with or without inhibitors. Following incubation, TB was discarded, and the cells were washed three times with 100 μL of ice-cold TB for each well to completely remove the residual incubation solution. After washing, 100 μL of distilled water was added to each well, and the cells were completely lysed by repeatedly freezing and thawing with liquid nitrogen for three cycles (−196°C to 37°C). Cell lysate (30 μL) was obtained and mixed with ice-cold methanol containing IS to precipitate proteins at a volume ratio of 1:4. The mixture was centrifuged at 12,000 rpm at 4°C for 5 min, and the supernatant was collected and used to determine the amount of the compounds and substrates using LC-MS. An additional 25 μL of cell lysate was used to determine the total protein concentration using a BCA protein assay kit.

Briefly, BSA standards were prepared and diluted to obtain concentrations of 25–2000 μg/mL. BCA working reagent (WR) was prepared by mixing 50 parts of BCA reagent A with 1 part of BCA reagent B (50:1, Reagent A: B). Subsequently, 25 μL cell lysate or BSA standard was added to 200 μL WR, followed by incubation for 30 min at 37°C. Finally, the absorbance of these samples was measured at 570 nm using a plate reader.

### 2.4 Transporter inhibition study using HEK293 cells

The procedures for cell culture, dosing solution preparation, cell lysate preparation, and sampling were consistent with those in the substrate study described above, except for the pre-incubation and incubation steps. For the OATP1B1 and OATP1B3 inhibition experiments, after the cells were washed twice with TB and incubated with TB for 5 min, 50 μL of the pre-dosing solution of the compounds or inhibitors at different concentrations were added into each assigned well for a 30-min incubation. The pre-dosing solution was subsequently discarded and replaced with 50 μL of the probe substrate and compounds or known inhibitor mixture incubated for 10 min at 37°C. Finally, the amount of substrate was quantified using LC-MS.

### 2.5 MRP2 substrate study using vesicle membranes

The substrate study was performed at 37°C with a water bath using a mixture comprising of 10 μL of vesicle membranes (5 mg/mL) and 10 μL of Buffer A2 (replaced with 5 μL Reagent G and 5 μL inhibitors for the inhibitor groups). The mixture was pre-incubated at 37°C with a water bath for 5 min. Meanwhile, 10 μL of dosing solution of the compounds or probe substrates and 10 μL of Reagent C2 (ATP) or D2 (AMP, as the non-ATP dependent control) were mixed and pre-incubated at 37°C for 5 min. Following pre-incubation, the two mixtures were mixed and incubated for an additional 5 min.

After incubation, the reactions were stopped with ice-cold Buffer B, filtered through a 96-well filter plate to separate vesicle-associated ligands from the matrix, and washed five times with ice-cold Buffer B. The vesicles on the filer plate were dissolved using 50 μL of 80% methanol (v/v), and the filtrate was collected by centrifugation at 2000 rpm for 2 min. The dissolution and centrifugation procedures were repeated. The filtrates were combined up to a total of 100 μL and mixed with 200 μL of ice-cold methanol containing IS, followed by centrifugation at 12,000 rpm for 5 min. The supernatant was collected to quantify the amount of compound or substrate using LC-MS.

### 2.6 MRP2 inhibition study using vesicle membranes

The incubation, termination, washing, and sampling steps were similar to those in the substrate study described above, except for the mixture content. Briefly, 10 μL of vesicle membranes and 5 μL of Reagent G were mixed and pre-incubated with 10 μL of the compounds or Buffer A2 (used as no compound-treatment control [NC]) at 37°C for 5 min. Meanwhile, 5 μL of MPAG and 20 μL of Reagent C2 (ATP) or D2 (AMP, used for non-ATP dependent control) were mixed and pre-incubated at 37°C for 5 min. The two mixtures were mixed before the incubation and then incubated for an additional 5 min. Finally, the supernatant was collected to analyze the amount of MPAG using LC-MS.

### 2.7 LC-MS analysis

The LC-MS system used in this study contained a LEAP CTC auto sampler (HTS PAL), liquid chromatograph (LC 20AD), and triple-quadrupole mass spectrometer (API 4000), and was programmed using the Analyst^®^1.6.3 software. The detailed conditions for the LC-MS analysis of glucuronide formation, transporter substrates, and MPAG are shown in Supplementary Texts S1–S3 and Supplementary Table S1.

### 2.8 Data analysis

#### 2.8.1 Data analysis of UGT induction

The formation of glucuronide metabolites, as determined with LC-MS, was used to calculate the formation rate linked to enzyme activity according to Equation 1.

$$\text{Formation rate }\left(\text{pmol}/10^{6}\text{ cells}/\min\right)=\frac{\text{amount}\;\text{of}\;\text{glucuronide}\;\text{metabolites}\;\text{formation}\;\left(\text{pmol}\right)}{\text{number}\;\text{of}\;\text{cells}\;\left(10^{6}\;\text{cells}\right)\times \text{incubation}\;\text{time}\;\left(\min\right)}$$ (1)

The percentage of enzyme activity relative to the NC (%) was determined using Equation 2.

$$\text{Relative enzyme activity }\left(\%\text{ of NC}\right)=\frac{\text{formation}\;\text{rate}\;\text{in}\;\text{the}\;\text{compound}-\text{treated}\;\text{group}}{\text{that}\;\text{in}\;\text{NC}}\times 100$$ (2)

#### 2.8.2 Data analysis of solute carrier (SLC) substrate and inhibition study

Regarding the substrate assay, the concentrations of the compounds or probe substrates in the cell lysate were determined using LC-MS and used to calculate the uptake rate (pmol/mg/min) using Equation 3.

$$\text{Uptake rate }\left(\text{pmol}/\text{mg}/\min\right)=\frac{C_{lystate}}{P\times T}$$ (3)

where, C_lysate_, P, and T represent the concentrations detected in the cell lysate (pmol), protein concentration in the cells (mg), and incubation time (min), respectively.

The UR was calculated using Equation 4.

$$\text{UR}=\frac{\text{average}\;\text{of}\;\text{uptake}\;\text{rate}\;\text{in}\;\text{transporter}-\text{expressing}\;\text{cells}}{\text{that}\;\text{in}\;\text{MOCK}\;\text{cells}}$$ (4)

Finally, the effect of known inhibitors on the uptake rate was evaluated using the inhibition rate (%), which was calculated using Equation 5.

$$\text{Inhibition rate }\left(\%\right)=\left(1-\frac{\left(\text{uptake}\;\text{rate}\;\text{in}\;\text{transporter}-\text{expressing}\;\text{cells}\;\text{with}\;\text{inhibitor}\right)-\left(\text{that}\;\text{in}\;\text{MOCK}\;\text{cells}\;\text{with}\;\text{inhibitor}\right)\;}{\left.\text{uptake}\;\text{rate}\;\text{in}\;\text{transporter}-\text{expressing}\;\text{cells}\;\text{without}\;\text{inhibitor})-(\text{that}\;\text{in}\;\text{MOCK}\;\text{cells}\;\text{without}\;\text{inhibitor}\right)}\right)\times 100$$ (5)

If the UR was ≥2.0, and the inhibition rate was ≥50% after treatment with a known transporter inhibitor, the compound was considered a substrate for the SLC transporter.

For the inhibition assay, the concentrations of substrates in the cell lysate treated with different concentrations of inhibitors were determined *via* LC-MS, and the calculation methods for the uptake rate and UR were similar to those in the substrate assay. The net uptake rate of the substrate (pmol/mg/min), namely, transporter-mediated uptake, was calculated using Equation 6.

$$\text{Net uptake rate}=\left(\text{uptake rate of substrate in transporter}-\text{expressing cells}\right)-\left(\text{average of that in MOCK cells}\right)$$ (6)

Subsequently, the changes in transporter activity were expressed as percentages of the net uptake rate relative to NC, arbitrarily set at 100%, according to Equation 7.

$$\text{Relative net uptake rate }\left(\%\text{ of NC}\right)=\frac{\text{net}\;\text{uptake}\;\text{rate}\;\text{in}\;\text{the}\;\text{compounds}-\text{treated}\;\text{group}}{\text{average}\;\text{of}\;\text{that}\;\text{in}\;\text{NC}}\times 100$$ (7)

The potential inhibitory effect of the compounds on transporter activity was further evaluated using the half-maximal inhibitory concentration (IC_50_). IC_50_ and 95% confidence intervals were calculated using GraphPad Prism 8 software (GraphPad Software, Inc., San Diego, USA), through nonlinear regression based on a three-parameter logistic function using Equation 8.

$$Y=\frac{100}{1+10^{X-{\text{LogIC}}_{50}}}$$ (8)

Where X represents the concentration of compounds (inhibitors), and Y represents the relative net uptake rate.

The potential of the compound to inhibit OATP1B1 and OATP1B3 expression determined by calculating the R value using Equation 9. According to the DDI guidelines, a compound has the potential to inhibit OATP expression *in vivo* if the R value is more than 1.1.

$$R=1+\frac{f_{u,p}\times I_{in,\max}}{{IC}_{50}}$$ (9)

Where f_u,p_ is the unbound fraction of the compound (inhibitor) in the plasma, IC_50_ is the half-maximal inhibitory concentration, and I_in,max_ is the estimated maximum plasma compound concentration at the liver inlet, which was calculated using Equation 10.

$$I_{in,\max}=I_{\max}+\frac{F_{a}\times F_{g}\times k_{a}\times \text{Dose}}{Q_{h}\times R_{B}}$$ (10)

Where F_a_ is the fraction of the compound (inhibitor) absorbed, F_g_ is the intestinal availability, k_a_ is the absorption rate constant, Q_h_ is the hepatic blood flow rate, which is determined as 97 L/h (70 kg body weight), and R_B_ is the blood-to-plasma concentration ratio.

#### 2.8.3 Data analysis of MPR2 substrate and inhibition study

For the substrate assay, the amount of compounds in the vesicle was determined with LC-MS to calculate the transport activity, UR, and inhibition rate using Equations 11–13, which were used to determine whether the compounds were substrates of the transporter.

$$\text{Transported activity }\left(\text{pmol}/\min/\text{mg}\right)=\frac{\left(\text{amount}\;\text{of}\;\text{compounds}\;\text{in}\;\text{vesicles}\;\text{driven}\;\text{by}\;\text{ATP}-\text{that}\;\text{in}\;\text{vesicles}\;\text{driven}\;\text{by}\;\text{AMP}\right)\left(\text{pmol}\right)}{\text{incubation}\;\text{time}\;\left(\min\right)\times \text{vesicles}\;\text{protein}\;\left(\text{mg}\right)}$$ (11)

$$\text{UR}=\frac{\text{average}\;\text{amount}\;\text{of}\;\text{compounds}\;\text{in}\;\text{vesicles}\;\text{driven}\;\text{by}\;\text{ATP}}{\text{that}\;\text{in}\;\text{vesicles}\;\text{driven}\;\text{by}\;\text{AMP}}$$ (12)

$$\text{Inhibition rate }\left(\%\right)=\left(1-\frac{\text{uptake}\;\text{ratio}\;\text{of}\;\text{compounds}\;\text{in}\;\text{the}\;\text{presence}\;\text{of}\;\text{inhibitors}}{\text{that}\;\text{in}\;\text{the}\;\text{absence}\;\text{of}\;\text{inhibitors}}\right)\times 100$$ (13)

If the UR was ≥2.0, and the inhibition rate was ≥50% after being treated with a known transporter inhibitor, the compound was considered as a substrate of the MRP2 transporter.

Regarding the inhibition assay, the methods used to calculate the transported activity and UR were similar to those used for the substrate assay. However, please refer to the SLC inhibition assay for the equations for calculating the UR percentages relative to the NC and IC_50_ values of compounds on MRP2 activity and MRP2-mediated MPAG transport.

#### 2.8.4 Statistical analysis

All experiments were performed in triplicate (N = 3) for each group, except for those for determining the inhibitory effect of seven drugs on transporters, which were performed in duplicated (N = 2). The *p*-value calculated with EXCEL (Microsoft, Redmond, WA) using a *t*-test was used to determine which treatment group was statistically different from the vehicle or negative control.

## 3 Results

### 3.1 Prednisolone promotes the metabolism of MPA to MPAG by inducing UGT activity

The glucuronidation of MPA is primarily known to occur in the liver and, to a certain extent, in the gastrointestinal tract and kidney. UGT1A8, UGT1A9, UGT1A10, and UGT2B7 are involved in the metabolism of MPA to MPAG. UGT1A9 and UGT2B7 are considered the major metabolizing enzymes of MPA owing to their relatively high hepatic and renal expression.

After treating human hepatocytes with prednisolone, the active metabolite of prednisone, and CsA at concentrations ranging from 0.5 to 100 μM for 96 h, the incubation medium was replaced with 100 μM 4-trifluoromethyl-7-hydroxycoumarin and 100 μM β-estradiol, which are probe substrates of UGT1A9/2B7 and UGT1A1/1A3, respectively, for an additional incubation of 30 min. The formation of 4-trifluoromethyl-7-hydroxycoumarin glucuronide and estradiol-3-glucuronide was detected to evaluate the induction of UGT enzyme activity compared with that in the NC group. In this study, omeprazole, which is a known UGT inducer, was used as the positive control (Schaefer et al., 2012).

**FIGURE 1:**
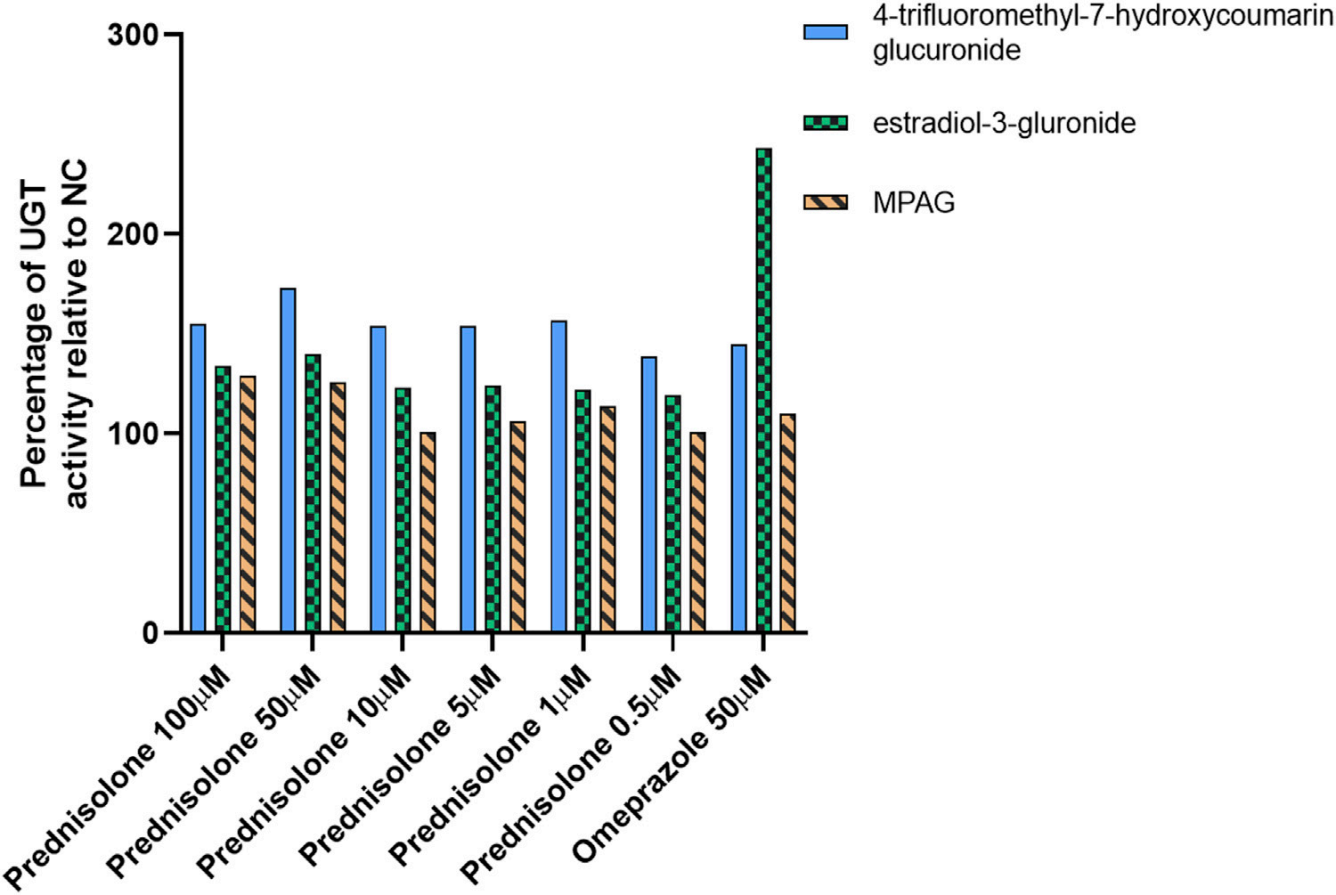
Inducible effect of prednisolone on UGT activity in human hepatocytes. LC-MS, liquid chromatography-mass spectrometry; MPA, mycophenolic acid; MPAG, mycophenolic acid glucuronide; NC, non-treatment control; UGT, uridine 5′-diphospho-glucuronosyltransferase.

After treatment with prednisolone at concentrations of 0.5, 1, 5, 10, 50, and 100 μM, the glucuronidation activity of 4-trifluoromethyl-7-hydroxycoumarin in hepatocytes was 139%, 157%, 154%, 154%, 173%, and 155% of that in the NC group, respectively (*p*-value <0.05), and the β-estradiol glucuronidation activity in hepatocytes was 119%, 122%, 124%, 123%, 140%, and 134% of that in the NC group, respectively (Figure 1 and Supplementary Table S2, *p*-value <0.05). After treatment with omeprazole, the glucuronidation activities of 4-trifluoromethyl-7-hydroxycoumarin and β-estradiol were 145% and 243%, respectively (*p*-value <0.05). Furthermore, the conversion of MPA to MPAG in human hepatocytes was evaluated after incubation with prednisolone for 96 h. A slight increase in MPAG formation was observed in the prednisolone-treated groups.

These results suggest that prednisolone can significantly induce UGT activity, particularly the UGT1A9 and UGT2B7 isoforms, resulting in increased metabolism of MPA to MPAG. However, this induction potential was not observed in CsA-treated human hepatocytes (data not shown).

### 3.2 CsA inhibits transport of MPAG mediated by OATP1B1 and OATP1B3

First, we examined the UR of MPAG at concentrations of 0.5, 1, and 10 μM after incubation with HEK293 cells expressing human OATP1B1 or OATP1B3 to determine whether MPAG uptake is mediated by OATP1B1 and OATP1B3 transporters. The inhibition rate of MPAG was determined after treatment with rifampicin, which is a selective inhibitor of OATP1B1 and OATP1B3.

**FIGURE 2:**
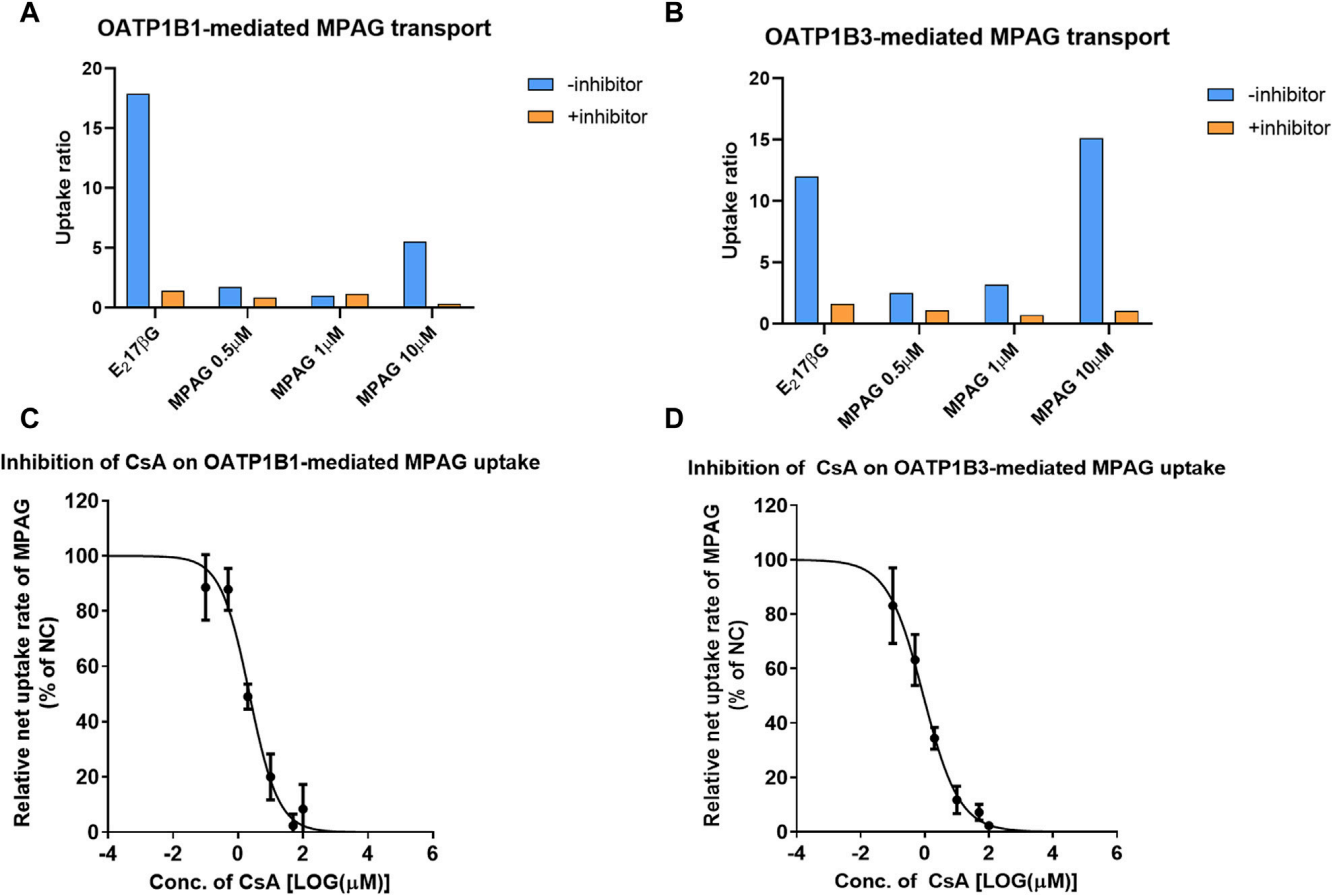
Inhibitory effect of CsA on MPAG uptake in OATP1B1- and OATP1B3-expressing cells. **(A, B)** illustrate the uptake ratio of MPAG and the probe substrate E_2_17βG in OATP1B1- and OATP1B3-expressing cells, respectively. **(C, D)** demonstrate the inhibitory effect of CsA on the uptake of MPAG mediated by OATP1B1 and OATP1B3, respectively. CsA, cyclosporine; MPAG, mycophenolic acid glucuronide; OATP, organic anion-transporting polypeptide; E_2_17βG, estradiol-17β-glucuronide.

At a concentration of 10 μM, the UR of MPAG mediated by OATP1B1 and OATP1B3 was 5.54 and 15.1, respectively, which decreased by 100% and 99.7% after being treated with 100 μM rifampicin (Figure 2; Supplementary Table S3). In contrast, the URs of estradiol-17β-glucuronide (E_2_17βG), used as a probe substrate of OATP1B1 and OATP1B3, were 17.9 and 12.1, respectively, and were inhibited by 97.7% and 92.7%, respectively, after rifampicin treatment. Therefore, these results suggest the potential of MPAG as a substrate for OATP1B1 and OATP1B3, consistent with observations reported by previous studies (Matsunaga et al., 2014).

We then investigated the concentration-dependent inhibitory effects of prednisolone and CsA on the uptake activity of 10 μM MPAG mediated by OATP transporters. CsA, rather than prednisolone, was shown to have potential inhibitory effects on the OATP-mediated transport of MPAG. The IC_50_ of CsA was estimated to be 2.25 and 0.906 μM for OATP1B1 and OATP1B3, respectively. However, the IC_50_ of rifampicin on MPAG uptake was 0.528 and 0.224 μM for OATP1B1 and OATP1B3, respectively. CsA inhibited OATP-mediated MPAG uptake, and its inhibitory potency was comparable to that of rifampicin.

Finally, the R value was calculated to assess the clinical risk of CsA inhibiting OATP-mediated MPAG uptake. At 300 mg dosage, the maximum plasma concentration of CsA at the liver inlet was 1924 ng/mL. The R value of CsA inhibiting OATP1B1 and OATP1B3-mediated transport of MPAG was 1.05 and 1.13, respectively, indicating that CsA has the potential to inhibit OATP1B3-mediated transport of MPAG. The detailed parameters are shown in Supplementary Table S4.

### 3.3 Prednisolone and CsA do not inhibit MRP2-mediated MPAG efflux

**FIGURE 3:**
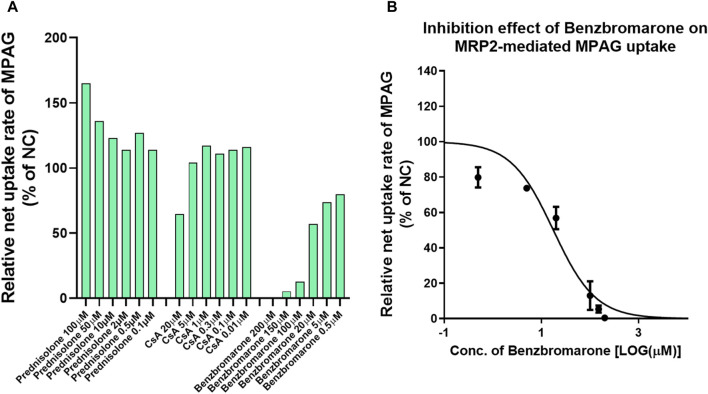
Inhibitory effect of CsA and prednisolone on MPAG uptake in MRP2-expressing membrane vesicles. **(A)** Illustrates the relative uptake activity of MPAG by MRP2 following incubation with different concentrations of CsA, prednisolone, and a known selective inhibitor, respectively, compared to the non-treatment group (set as NC). **(B)** Demonstrates the inhibitory effect of benzbromarone on the MRP2-mediated uptake of MPAG. CsA, cyclosporine; MPAG, mycophenolic acid glucuronide; MRP2, multidrug resistance-associated protein 2; NC, non-treatment group.

Several studies have demonstrated that MRP2 is involved in the renal and biliary excretion of MPAG *in vivo*. We evaluated the inhibitory potential of prednisolone and CsA on human MRP2 transporters using membrane vesicles. The UR of 2 μM MPAG was 18.8, and a known inhibitor, benzbromarone, demonstrated apparent inhibitory effects on MPAG uptake mediated by MRP2 with an IC_50_ of 17.6 μM (Figure 3). However, such inhibition was not detected after treatment with prednisolone and CsA.

### 3.4 MPAG is a weak substrate of OAT3

MPAG has been shown to be transported by OAT3; however, the study only evaluated the transport rate in human OAT3-expressing cells compared with empty vector-expressing cells, as well as the changes following incubation with inhibitors. Therefore, we further determined the UR of MPAG mediated by human OAT1 and OAT3 as well as the change in the UR following treatment with probenecid, a known selective inhibitor of OAT1 and OAT3.

**FIGURE 4:**
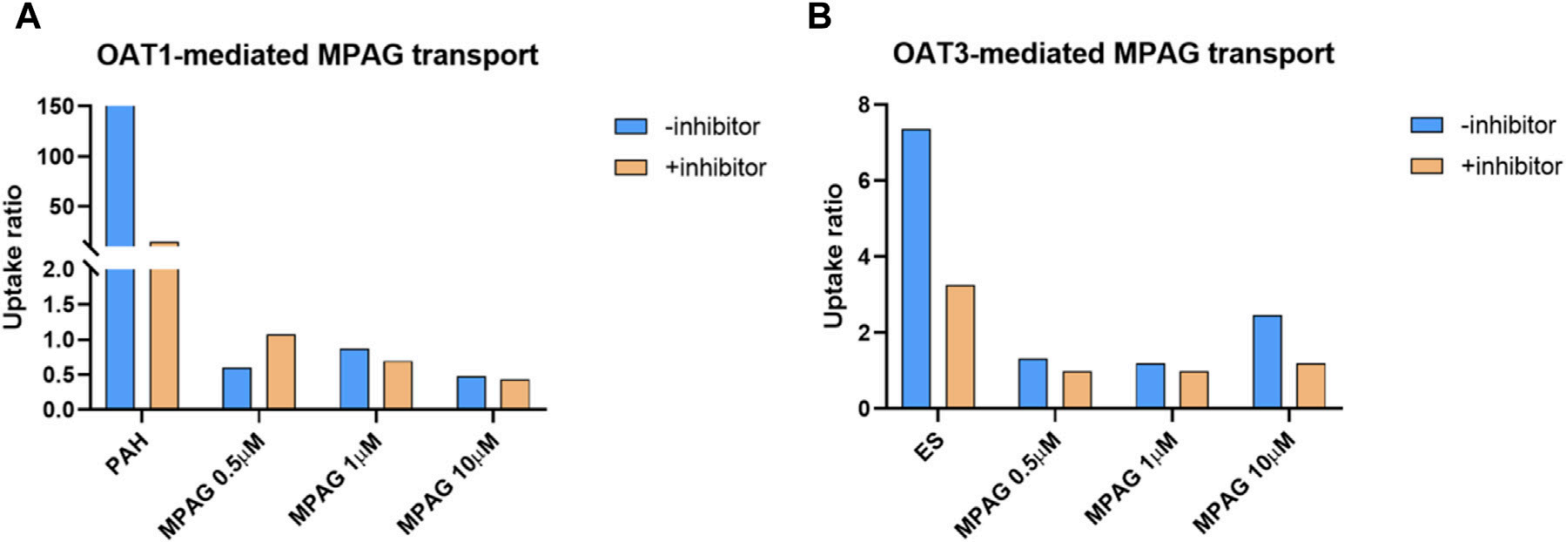
Substrate study of MPAG in OAT-expressing cells. **(A, B)** demonstrate the uptake ratio of MPAG and the probe substrate PAH (for OAT1) or ES (for OAT3), with or without inhibitor, in OAT1-and OAT3-expressing cells, respectively. MPAG, mycophenolic acid glucuronide; OAT, organic anion transporter; PAH, p-aminohippuric acid; ES, estrone-3-sulfate.

The UR of p-aminohippuric acid (PAH), used as a probe substrate for OAT1, in OAT1-expressing cells compared to that in MOCK cells was 159, which decreased to 14.6 (91.4% inhibition) following probenecid treatment (Figure 4). At concentrations of 0.5, 1, and 10 μM, the URs of MPAG mediated by OAT1 were all less than 2.0, indicating that MPAG is not a substrate of OAT1.

In the OAT3 substrate assay, the UR of the probe substrate estrone-3-sulfate (ES) was 7.37, which decreased to 3.26 (69.4% reduction) after probenecid treatment. At a concentration of 10 μM, the UR of MPAG mediated by OAT3 was 2.47, which is close to the reference value and thereby considered a substrate. Following incubation with probenecid, the UR of MPAG decreased to 1.19 (90.8% reduction). Therefore, this result suggests that MPAG is a weak substrate for OAT3.

### 3.5 *Salvia miltiorrhiza* inhibits OATP and OAT3 activity

The results of our study indicated that MRP2, OAT3, OATP1B1, and OATP1B3 are responsible for MPAG efflux or influx. Based on clinical practice in China, the inhibitory effects of seven drugs that are usually co-administered with MMF (cefoperazone, pantoprazole, meropenem, with probenecid, alprostadil, ganciclovir, *S. miltiorrhiza*, and olaparib) were evaluated for their impact on the activity of the abovementioned transporters.

**FIGURE 5:**
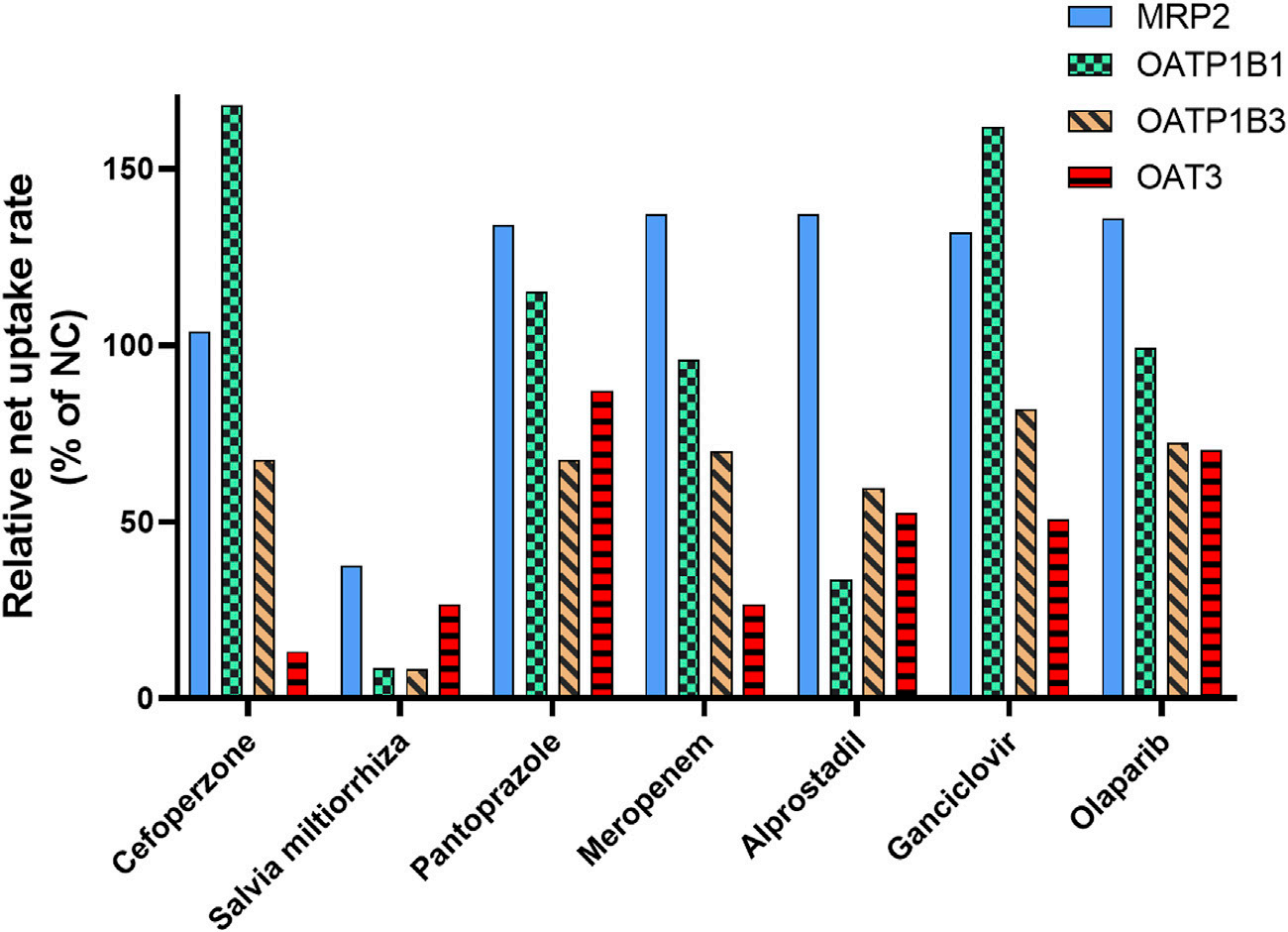
Inhibitory effect of the co-administrated drugs on the activity of the transporter involved in MPAG uptake. E_2_17βG, estradiol-17β-glucuronide; ES, estrone-3-sulfate; LC-MS, liquid chromatography-mass spectrometry; MMF, mycophenolate mofetil; MPAG, mycophenolic acid glucuronide; MRP2, multidrug resistance-associated protein 2; OATP, organic anion-transporting polypeptide.

No apparent inhibitory effect on MRP2 was observed following treatment with 50 μM or 50 μg/mL of the seven drugs (Figure 5 and Supplementary Table S5), suggesting that these drugs did not inhibit the activity of MRP2. For OATP1B1, following incubation with 50 μM *S. miltiorrhiza* and alprostadil, its activity was found to be reduced to 8.67% and 33.6% relative to NC, respectively. Regarding OATP1B3, following treatment with 50 μM *S. miltiorrhiza*, its activity decreased to 8.33% of that in NC. Following treatment with 50 μM of cefoperazone, *S. miltiorrhiza*, and meropenem, the transport activity of OAT3 decreased to 13.3%, 26.5%, and 26.6% of that observed in NC, respectively.

Therefore, these results indicate a potential DDI risk between *S. miltiorrhiza* and MMF. *Salvia miltiorrhiza* showed apparent inhibitory effects on OATP and OAT3 transporters, which are involved in hepatic uptake and renal excretion of MPAG. Furthermore, *S. miltiorrhiza* reportedly has an inhibitory effect on OAT1 and OAT3 and is considered a substrate of OATP1B1 and OATP1B3 (Wang and Sweet, 2012; Lu et al., 2022). However, no reports exist on its inhibitory effect on OATP transporters.

## 4 Discussion

A triple immunosuppressive regimen, including MMF, TAC, or CsA, with or without corticosteroids, is commonly used to prevent allograft rejection after solid organ transplantation. We conducted a systematic *in vitro* evaluation of metabolism- and transporter-based DDIs between MMF and co-administered medications to analyze the potential influence of co-medications on the high individual variability in MMF exposure. Our findings may facilitate the further development of mechanistic MMF PK models and individualized therapies.

Although various preclinical studies have shown that corticosteroids can influence MPA PK by inducing the expression of UGTs, altering the expression of MRP2 and OAT3, or displacing protein binding, options for steroid-MPA PK interactions remain contradictory. Our *in vitro* analysis in human hepatocytes revealed that prednisolone can potentially induce the activity of UGTs, particularly the UGT1A9 and UGT2B7 isoforms, resulting in the increased metabolism of MPA to MPAG. However, these induction potentials were undetected in CsA-treated human hepatocytes, which highlights the difference in steroid-MPA PK interactions between CsA- and TAC-based triple immunosuppressive regimens. Therefore, the effects of steroid tapering or withdrawal on the MPA PK levels should also be considered based on the co-administration of TAC.

*In vitro* results also showed that CsA, rather than prednisolone, inhibits OATP-mediated transport of MPAG, which may reduce the subsequent reabsorption of MPA through EHC (van Gelder, 2021). Moreover, CsA and prednisolone showed no inhibitory effect on MRP2, which is responsible for MPAG excretion into the bile. Geng *et al.* reported lower MPA and higher MPAG area under the curve in patients receiving triple therapies with MMF, CsA, and prednisone (Geng et al., 2012). This phenomenon may be mainly attributed to the concentration-dependent inhibition of CsA on the OATP transporter-mediated uptake activity of MPAG. Multivariate analysis by Geng *et al.* indicated a lower MPAG area under the curve in the presence of prednisone, and steroid-induced transporters may contribute to the enhancement of MPAG elimination (Geng et al., 2012). However, no interaction between prednisolone and MRP2-mediated MPAG efflux was observed *in vitro* in this study.

Although MPAG is reported to be likely transported by OAT3, the study only evaluated the transport rate in human OAT3-expressing cells compared to empty vector-expressing cells and its change following incubation with inhibitors (Uwai et al., 2007). OAT1 is also a major transporter involved in renal excretion. Therefore, this study determined the UR of MPAG mediated by human OAT1 and OAT3, and the change in the UR after probenecid treatment, which is a known selective inhibitor of OAT1 and OAT3. The results showed that the UR of MPAG mediated by OAT3 was close to the cut-off value for substrate determination, and its UR decreased by 90.8% after incubation with probenecid, indicating that MPAG is a weak substrate of OAT3, which is consistent with previous results (Uwai et al., 2007). Furthermore, other immunosuppressants did not affect OAT3 expression.

In addition to the DDIs between the triple immunotherapies, the impact of seven commonly co-administered drugs on the MMF PK pathway was evaluated. *Salvia miltiorrhiza* inhibited OATP and OAT3 transporters, affecting hepatic uptake and renal excretion of MPAG, resulting in increased MPAG exposure. *Salvia miltiorrhiza* is a traditional Chinese medicine used to treat patients with cardiovascular disease (Zhou et al., 2005). Wang *et al.* reported that *S. miltiorrhiza* injections strongly inhibit OAT1 and OAT3 functions *in vivo* (Wang et al., 2014). It is hypothesized that certain components of *Salvia miltiorrhiza* may be substrates of these transporters, which requires further investigation. Lu *et al.* reported lithospermic acid and salvianolic acid B as substrates of OATP1B1 and OATP1B3, and protocatechuic acid and tanshinol as substrates of OAT1 (Lu et al., 2022). However, caution should be exercised regarding the potential DDIs of herbal medicines. Furthermore, *S. miltiorrhiza* may increase free drug concentrations by competitively binding to human serum albumin (Shao et al., 2016).

Although the steroid-MPA DDI has been demonstrated *in vitro*, the clinical significance of this interaction remains controversial. Rong *et al.* conducted a comprehensive analysis of the clinical evidence pertaining to steroid-MPA PK interaction and found 8 *supporting* and 22 *non-supporting* papers for purported drug interactions (Rong and Kiang, 2023). The study design, including the sample size, sampling scheme, statistical analysis methods, and form of prednisone/prednisolone investigated as a covariate, may have resulted in potential false-negative findings in *non-supporting* papers. Additionally, differences between CsA- and TAC-based regimens may also contribute to clinical outcomes, providing useful insights in guiding the design of future clinical DDI studies.

Because not all studies can be evaluated in a well-controlled manner, population PK (popPK) analysis may be the sole method to assess potential DDIs. These models are usually based on a static- or physiologically based dynamic approach (Perdaems et al., 2010). The perpetrator of DDIs was identified as a categorical covariate in popPK models using a static approach. However, dynamic and concentration-dependent inhibition/induction were not analyzed. Compared to the static approach, the dynamic semi-physiologically based population PK model approach can consider time-varying inhibition/induction processes and explore sources of unexplained inter-individual variability (IIV) based on *in vivo* data (Ashraf et al., 2018).

Regarding the irrational study design, the potential sudden change in plasma concentrations due to DDIs could be interpreted as inter-occasion variability. Furthermore, data quality and patient compliance may also affect PK parameter estimates (Bonate et al., 2016). However, no standard sample size exists for popPK studies conducted to identify the fixed effects of DDIs. IIV and residual variability may influence the evaluation of PK interactions. Therefore, the application of popPK modeling with optimal design and simulation may prove useful in determining the sample size and power of a DDI study (Yang and Beerahee, 2011).

Our study had some limitations. First, the induction time *in vitro* was relatively short and could not fully simulate repeated clinical drug administration. Second, the influence of formulations (i.e., MMF and enteric-coated mycophenolate sodium), which may have determined the site of DDI action, was not considered. Third, pharmacodynamic interactions were not analyzed in this study.

In conclusion, metabolism- and transporter-based DDIs between MMF and co-administered medications were systematically evaluated *in vitro*. Prednisolone promoted the metabolism of MPA to MPAG by inducing UGT activity in TAC-based triple immunotherapy. CsA inhibited the transport of MPAG mediated by OATP1B1 and OATP1B3. *Salvia miltiorrhiza* showed apparent inhibitory effects on OATP and OAT3 transporters, which are involved in hepatic uptake and renal excretion of MPAG. Therefore, these factors may contribute to the high IIV in MMF exposure and facilitate further development of mechanistic MMF PK models and individualized therapies.

## Data Availability

The raw data supporting the conclusions of this article will be made available by the authors, without undue reservation.

## Supplementary material

SUPPLEMENTARY TEXT S1 LC-MS Analysis of Glucuronide Formation.

SUPPLEMENTARY TEXT S2 LC-MS Analysis of Probe Substrate of Transporters.

SUPPLEMENTARY TEXT S3 LC-MS Analysis of MPAG.

SUPPLEMENTARY TABLE S1 List of the analytes and the corresponding multiple reaction monitoring parameters.

SUPPLEMENTARY TABLE S2 Inducible effect of prednisolone on UGT activity in human hepatocytes.

SUPPLEMENTARY TABLE S3 Inhibitory effect of prednisolone, CsA and rifamycin on the uptake of MPAG mediated by OATP1B1 and OATP1B3.

SUPPLEMENTARY TABLE S4 The parameters for calculating the R value of CsA inhibiting the OATP-mediated MPAG uptake.

SUPPLEMENTARY TABLE S5 Inhibitory effect of seven drugs on the activity of MRP2, OAT3, OATP1B1 and OATP1B3.
