# Supplementary material for: In vitro mechanistic study on mycophenolate mofetil drug interactions: effect of prednisone, cyclosporine, and others
Source: Front Pharmacol. 2024 Aug 26;15:1443794. doi: 10.3389/fphar.2024.1443794 (PMC11381307; doi:10.3389/fphar.2024.1443794)
Supplement: Supplementary file 1 [file DataSheet1.docx]

# Electronic Supplementary Material

**Supplementary Text S1 LC-MS Analysis of Glucuronide Formation**

The enzyme activity of different UGT isoforms was determined by the formation of glucurondie metabolites including 4-trifluoromethyl-7-hydroxycoumarin glucuronide and estradiol-3-gluronide, of which the chromatographic separation was achieved using gradient conditions on a Phenomenex Synergi 4 um Hydro RP 30*2 mm 80 Å HPLC column. The mobile phases consisted of 0.1% (v/v) formic acid in water (A) and 0.1% (v/v) formic acid in acetonitrile (B) at a flow rate of 450 μL/min. The injection volume was 5 μL, and the column temperature was maintained at 40°C. The LC conditions for the analysis of the above two metabolites were the same, which was set as: t=0.00 min, %B=10; t=0.50 min, %B=95; t=1.00 min, %B=95%; t=1.01 min, %B=95; t=1.30 min, %B=10. The ionized molecules were detected multiple reaction monitoring (MRM) in negative mode. The detailed parameters including IS are stated in Supplementary Table S1.

**Supplementary Text S2 LC-MS Analysis of Probe Substrate of Transporters**

Estradiol-17β-D-glucuronide (E_2_17βG), P-aminohippuric acid (PAH) and Estrone-3-sulfate (ES) were used as the probe substrate of OATP1B1, OAT1 and OAT3, respectively, in this study. For quantification of these substrates, chromatographic separation was performed on a Phenomenex Synergi 4 um Hydro RP 30*2 mm 80 Å HPLC column at a temperature of 40°C. The flow rate was 450 μL/min. The injection volume was 5 μL. Mobile phase A consisted of water with 0.1% (v/v) formic acid, and mobile phase B consisted of acetonitrile with 0.1% (v/v) formic acid. The gradient elution for the analysis of E_2_17βG was set as: t=0.00 min, %B=10; t=0.50 min, %B=95; t=1.50 min, %B=95%; t=1.51 min, %B=10; t=2.50 min, %B=10. The gradient elution for the analysis of both PAH and ES was set as: t=0.00 min, %B=30; t=1.00 min, %B=95; t=1.50 min, %B=95%; t=1.51 min, %B=30; t=1.80 min, %B=30. Analytes of both E_2_17βG and ES were detected using MRM in negative mode, while for PAH, which was in positive mode. The detailed parameters are shown in Supplementary Table S1.

**Supplementary Text S3 LC-MS Analysis of MPAG**

Chromatographic separation for MPAG was obtained using a Phenomenex Synergi 4 um Hydro RP 30*2 mm 80 Å HPLC column. The mobile phase (solvent A) consisted of 0.1% (v/v) formic acid in water, whereas the organic phase (solvent B) consisted of 0.1% (v/v) formic acid in acetonitrile. MPAG was resolved using following gradient: t=0.01 min, %B=5; t=0.40 min, %B=75; t=0.41 min, %B=95%; t=0.80 min, %B=95; t=0.81 min, %B=5; t=1.10 min, %B=5. A total of run time was 1.10 min, the flow rate, injection volume and column temperature were 450 μL/min, 5 μL and 45ºC, respectively. The ionized molecules were detected multiple reaction monitoring (MRM) in positive mode. The detailed parameters including IS are stated in Supplementary Table S1.

**Supplementary Table S1 List of the analytes and the corresponding multiple reaction monitoring parameters**

| Substrate | MRM1 (Da) | MRM2 (Da) | DP (V) | EP (V) | CE (V) | CXP (V) | IS |
| --- | --- | --- | --- | --- | --- | --- | --- |
| MPAG | 495.5 | 319.0 | -56 | -9 | -26 | -16 | Tolbutamide |
| 4-trifluoromethyl-7-hydroxycoumarin glucuronide | 404.8 | 175.1 | -90 | -10 | -15 | -14 | Tolbutamide |
| estradiol-3-gluronide | 447.5 | 74.9 | -100 | -10 | -58 | -10 | Tolbutamide |
| E_2_17βG | 447.5 | 74.9 | -100 | -10 | -58 | -10 | Tolbutamide |
| PAH | 195.2 | 120.1 | 44 | 10 | 10 | 17 | Tolbutamide |
| ES | 249.1 | 269.0 | -90 | -10 | -40 | -16 | Tolbutamide |
| Tolbutamide | 269.3 | 170.0 | -55 | -10 | -25 | -25 | Negative mode |
| Tolbutamide | 271.1 | 155 | 57 | 10 | 22 | 15 | Positive mode |

MRM1, parention Q1 (Da); MRM2, production Q3 (Da); DP, declustering potential (V); EP, entrance potential (V); CE, collision energy (V); CXP, cell exit potential (V)

**Supplementary Table S2 Inducible effect of prednisolone on UGT activity in human hepatocytes**

| Test Conc (μM) | formation of 4-trifluoromethyl-7-hydroxycoumarin glucuronide (nM) | | | % of NC | | | | | P value (vs NC) |
| --- | --- | --- | --- | --- | --- | --- | --- | --- | --- |
|  | Sample1 | Sample2 | Sample3 | Sample1 | Sample2 | Sample3 | Mean | SD |  |
| NC | 949 | 961 | 868 | 102 | 104 | 93.7 | 100 | 5.46 | NA |
| Ome | 1050 | 1670 | 1310 | 113 | 180 | 141 | 145 | 33.6 | 0.0837 |
| 100 | 1310 | 1610 | 1380 | 141 | 174 | 149 | 155 | 16.9 | 0.0060 |
| 50 | 1690 | 1560 | 1550 | 183 | 168 | 167 | 173 | 8.43 | 0.0002 |
| 10 | 1530 | 1490 | 1260 | 165 | 161 | 136 | 154 | 15.7 | 0.0049 |
| 5 | 1680 | 1100 | 1510 | 181 | 119 | 163 | 154 | 32.2 | 0.0447 |
| 1 | 1610 | 1420 | 1330 | 174 | 153 | 144 | 157 | 15.4 | 0.0038 |
| 0.5 | 1280 | 1320 | 1270 | 138 | 143 | 137 | 139 | 2.86 | 0.0004 |
| Test Conc (μM) | formation of beta-estradiol glucuronide (nM) | | | % of NC | | | | | P value (vs NC) |
|  | Sample1 | Sample2 | Sample3 | Sample1 | Sample2 | Sample3 | Mean | SD |  |
| NC | 110 | 120 | 106 | 98.2 | 107 | 94.6 | 100 | 6.44 | NA |
| Ome | 268 | 271 | 278 | 239 | 242 | 248 | 243 | 4.58 | 0.00001 |
| 100 | 147 | 153 | 151 | 131 | 137 | 135 | 134 | 2.73 | 0.00106 |
| 50 | 171 | 147 | 154 | 153 | 131 | 138 | 140 | 11.0 | 0.00535 |
| 10 | 129 | 153 | 130 | 115 | 137 | 116 | 123 | 12.1 | 0.04620 |
| 5 | 146 | 134 | 137 | 130 | 120 | 122 | 124 | 5.58 | 0.00803 |
| 1 | 129 | 149 | 131 | 115 | 133 | 117 | 122 | 9.83 | 0.03286 |
| 0.5 | 135 | 134 | 132 | 121 | 120 | 118 | 119 | 1.36 | 0.00703 |
| Test Conc (μM) | formation of MPAG (nM) | | | % of NC | | | | | P value (vs NC) |
|  | Sample1 | Sample2 | Sample3 | Sample1 | Sample2 | Sample3 | Mean | SD |  |
| NC | 1140 | 1550 | 1300 | 85.7 | 117 | 97.7 | 100 | 15.5 | NA |
| Ome | 1170 | 1340 | 1890 | 88.0 | 101 | 142 | 110 | 28.3 | 0.6107 |
| 100 | 1780 | 1730 | 1630 | 134 | 130 | 123 | 129 | 5.74 | 0.0394 |
| 50 | 1750 | 1570 | 1690 | 132 | 118 | 127 | 126 | 6.89 | 0.0597 |
| 10 | 1530 | 1350 | 1160 | 115 | 102 | 87.2 | 101 | 13.9 | 0.9221 |
| 5 | 1490 | 1420 | 1300 | 112 | 107 | 97.7 | 106 | 7.22 | 0.6070 |
| 1 | 1480 | 1540 | 1540 | 111 | 116 | 116 | 114 | 2.60 | 0.1914 |
| 0.5 | 1380 | 1260 | 1380 | 104 | 94.7 | 104 | 101 | 5.21 | 0.9405 |

Note: after being treated by prednisolone with different concentrations, or treated by a known inducer omeprazole (Ome), the UGT activity was determined by detecting the formation of 4-trifluoromethyl-7-hydroxycoumarin glucuronide, beta-estradiol glucuronide and MPAG, respectively. The relative UGT activity (% of NC) was further determined by the formation rate of the metabolites in compound-treated group relative to that in no-treated control (used as NC and set as 100%). Statistical analysis of *P* value of treated groups compared to NC was preformed using t-test, when the *P* value less than 0.05 was typically considered to be statistically significant.

**Supplementary Table S3 Inhibitory effect of prednisolone, CsA and rifamycin on the uptake of MPAG mediated by OATP1B1 and OATP1B3**

| Inhibitory effect of prednisolone on the OATP1B1-mediated uptake of MPAG | | | | | | | | | |
| --- | --- | --- | --- | --- | --- | --- | --- | --- | --- |
| Test Conc (μM) | MPAG Conc. (nM) | | | Relative net uptake rate (% of NC) | | | | | IC_50_ value (μM) |
|  | Sample1 | Sample2 | Sample3 | Sample1 | Sample2 | Sample3 | Mean | SD |  |
| MOCK-NC | 9.03 | 14.6 | 15.5 | NA | NA | NA | NA | NA | 122 |
| NC | 34.3 | 41.4 | 36.5 | 86.0 | 117 | 96.9 | 100 | 15.8 |  |
| 100 | 25.8 | 31.2 | 22.8 | 52.3 | 74.2 | 41.2 | 55.9 | 16.8 |  |
| 50 | 24.2 | 36.2 | 34.5 | 59.8 | 93.2 | 86.7 | 79.9 | 17.7 |  |
| 10 | 35.2 | 36.8 | 36.4 | 83.7 | 94.9 | 93.9 | 90.9 | 6.22 |  |
| 2 | 38.1 | 42.4 | 41.2 | 94.0 | 117 | 112 | 108 | 12.2 |  |
| 0.5 | 37.3 | 44.5 | 40.7 | 94.0 | 125 | 111 | 110 | 15.6 |  |
| 0.1 | 37.9 | 49.8 | 81.7(OL) | 97.6 | 146 | NA | 122 | 34.2 |  |
| Inhibitory effect of CsA on the OATP1B1-mediated uptake of MPAG | | | | | | | | | |
| Test Conc (μM) | MPAG Conc. (nM) | | | Relative net uptake rate (% of NC) | | | | | IC_50_ value (μM) |
|  | Sample1 | Sample2 | Sample3 | Sample1 | Sample2 | Sample3 | Mean | SD |  |
| MOCK-NC | 4.91 | 5.79 | 7.92 | NA | NA | NA | NA | NA | 2.25 |
| NC | 34.4 | 33.1 | 32.6 | 105 | 99 | 96 | 100 | 4.40 |  |
| 20 | 8.22 | 5.40 | 11.0 | 7.50 | 0.00 | 17.7 | 8.41 | NA |  |
| 5 | 4.23 | 5.75 | 8.18 | 0.00 | 0.00 | 7.22 | 2.41 | 4.17 |  |
| 1 | 10.2 | 10.6 | 14.1 | 14.5 | 16.0 | 29.6 | 20.0 | 8.34 |  |
| 0.3 | 19.5 | 18.4 | 20.9 | 48.5 | 44.9 | 53.9 | 49.1 | 4.56 |  |
| 0.1 | 27.9 | 30.4 | 32.2 | 79.7 | 89.2 | 94.8 | 87.9 | 7.66 |  |
| 0.01 | 28.2 | 34.0 | 29.3 | 79.7 | 102 | 84.0 | 88.4 | 11.6 |  |
| Inhibitory effect of rifamycin on the OATP1B1-mediated uptake of MPAG | | | | | | | | | |
| Test Conc (μM) | MPAG Conc. (nM) | | | Relative net uptake rate (% of NC) | | | | | IC_50_ value (μM) |
|  | Sample1 | Sample2 | Sample3 | Sample1 | Sample2 | Sample3 | Mean | SD |  |
| MOCK-NC | 4.54 | 5.23 | 15.2 | NA | NA | NA | NA | NA | 0.528 |
| NC | 29.0 | 18.0 | 23.5 | 137 | 65.1 | 98.1 | 100 | 35.9 |  |
| 100 | 9.57 | 5.02 | 3.75 | 9.38 | 0.00 | 0.00 | 3.13 | 5.42 |  |
| 30 | 8.97 | 5.92 | 8.74 | 5.49 | 0.00 | 3.21 | 2.90 | 2.76 |  |
| 10 | 9.48 | 7.89 | 10.7 | 7.95 | 0.00 | 16.2 | 8.07 | 8.12 |  |
| 3 | 13.0 | 12.6 | 13.3 | 30.5 | 29.8 | 32.3 | 30.9 | 1.28 |  |
| 1 | 20.1(OL) | 11.8 | 13.3 | NA | 22.0 | 31.5 | 26.8 | 6.74 |  |
| 0.1 | 25.7 | 26.7 | 22.9 | 111 | 121 | 95.0 | 109 | 12.9 |  |
| Inhibitory effect of prednisolone on the OATP1B3-mediated uptake of MPAG | | | | | | | | | |
| Test Conc (μM) | MPAG Conc. (nM) | | | Relative net uptake rate (% of NC) | | | | | IC_50_ value (μM) |
|  | Sample1 | Sample2 | Sample3 | Sample1 | Sample2 | Sample3 | Mean | SD |  |
| MOCK-NC | 6.70 | 11.1 | 14.3 | NA | NA | NA | NA | NA | 119 |
| NC | 88.5 | 92.4 | 83.6 | 100 | 106 | 94.4 | 100 | 5.60 |  |
| 100 | 47.1 | 57.1 | 42.2 | 46.7 | 61.3 | 41.0 | 49.7 | 10.5 |  |
| 50 | 66.9 | 60.2 | 56.0 | 72.5 | 64.7 | 58.2 | 65.1 | 7.14 |  |
| 10 | 72.5 | 84.2 | 83.0 | 79.2 | 96.1 | 93.7 | 89.7 | 9.15 |  |
| 2 | 81.6 | 66.3 | 74.1 | 90.0 | 72.0 | 82.2 | 81.4 | 9.03 |  |
| 0.5 | 97.6 | 83.4 | 100.0 | 109 | 93.3 | 115 | 106 | 11.2 |  |
| 0.1 | 85.0 | 68.1 | 81.8 | 92.7 | 73.6 | 90.2 | 85.5 | 10.4 |  |
| Inhibitory effect of CsA on the OATP1B3-mediated uptake of MPAG | | | | | | | | | |
| Test Conc (μM) | MPAG Conc. (nM) | | | Relative net uptake rate (% of NC) | | | | | IC_50_ value (μM) |
|  | Sample1 | Sample2 | Sample3 | Sample1 | Sample2 | Sample3 | Mean | SD |  |
| MOCK-NC | 2.02 | 8.27 | 4.87 | NA | NA | NA | NA | NA | 0.906 |
| NC | 94.6 | 133(OL) | 70.7 | 115 | NA | 85.1 | 100 | 21.1 |  |
| 20 | 6.69 | 6.66 | 6.96 | 2.08 | 2.12 | 2.48 | 2.23 | 0.217 |  |
| 5 | 8.60 | 13.0 | 10.0 | 4.64 | 10.4 | 6.39 | 7.13 | 2.93 |  |
| 1 | 14.6 | 17.9 | 10.0 | 12.3 | 16.5 | 6.46 | 11.7 | 5.04 |  |
| 0.3 | 29.9 | 35.4 | 30.5 | 31.5 | 39.0 | 32.8 | 34.4 | 3.99 |  |
| 0.1 | 52.0 | 48.9 | 62.9 | 60.3 | 55.6 | 73.7 | 63.2 | 9.37 |  |
| 0.01 | 70.2 | 81.6 | 60.4 | 81.8 | 97.7 | 69.9 | 83.1 | 13.9 |  |
| Inhibitory effect of rifamycin on the OATP1B3-mediated uptake of MPAG | | | | | | | | | |
| Test Conc (μM) | MPAG Conc. (nM) | | | Relative net uptake rate (% of NC) | | | | | IC_50_ value (μM) |
|  | Sample1 | Sample2 | Sample3 | Sample1 | Sample2 | Sample3 | Mean | SD |  |
| MOCK-NC | 6.48 | 11.2 | 4.00 | NA | NA | NA | NA | NA | 0.224 |
| NC | 86.7 | 63.9 | 123(OL) | 116 | 83.9 | NA | 100 | 22.8 |  |
| 100 | 8.59 | 13.8 | 13.8 | 1.56 | 8.79 | 9.12 | 6.49 | 4.28 |  |
| 30 | 12.0 | 10.2 | 8.05 | 6.93 | 4.33 | 1.17 | 4.14 | 2.88 |  |
| 10 | 14.5 | 8.24 | 12.2 | 10.7 | 1.46 | 7.26 | 6.48 | 4.68 |  |
| 3 | 12.1 | 18.0 | 17.9 | 7.02 | 15.8 | 15.3 | 12.7 | 4.91 |  |
| 1 | 28.8 | 34.6 | 37.0 | 31.2 | 39.6 | 42.7 | 37.9 | 5.95 |  |
| 0.1 | 55.5 | 48.8 | 50.4 | 70.3 | 59.8 | 61.8 | 64.0 | 5.56 |  |

Note: the amount of MPAG transported by OATP1B1 and OATP1B3 was determined by LC-MS method. Then, the relative uptake rate of MPAG in the presence of either prednisolone, CsA or rifamycin was calculated by equation 7, finally, the IC_50_ value of inhibitory effect of CsA and rifamycin on OATP-mediated MPAG uptake was determined by GraphPad Prism software. OL means outlier, which was not used for calculation.

**Supplementary Table S4 The parameters for calculating the R value of CsA inhibiting the OATP-mediated MPAG uptake**

| **Paremeters** | **Values** | **Resource** |
| --- | --- | --- |
| Dose | 300 mg | / |
| f_u,p_ | 0.0727 | Legg and Rowland, 1988 |
| I_max_ | 974 ng/mL | / |
| k_a_ | 1.659 h^-1^ | Yoo S et al., 2019 |
| R_B_ | 1.62 | Yoo S et al., 2019 |
| F_a_ | 0.3 | Drugbank: https://go.drugbank.com/drugs/DB00091 |

F_a_, the fraction of the compound (inhibitor) absorbed; f_u,p_, the unbound fraction of the compound (inhibitor) in the plasma; I_in,max_, the estimated maximum plasma compound concentration at the liver inlet; k_a_, the absorption rate constant; R_B_, the blood-to-plasma concentration ratio.

Legg, B., Rowland, M., Saturable binding of cyclosporin A to erythrocytes: estimation of binding parameters in renal transplant patients and implications for bioavailability assessment. Pharmaceutical research 1988; (5): 80-85.

Yoon S, Yi S, Rhee SJ, et al. Development of a physiologically-based pharmacokinetic model for cyclosporine in Asian children with renal impairment. Transl Clin Pharmacol. 2019; 27(3):107-114.

**Supplementary Table S5 Inhibitory effect of seven drugs on the activity of MRP2, OAT3, OATP1B1 and OATP1B3**

| Test Comp. | Peak area of MRP2-mediated E_2_17βG uptake | | Relative net uptake rate (% of NC) | | |
| --- | --- | --- | --- | --- | --- |
|  | Sample1 | Sample2 | Sample1 | Sample2 | Mean |
| NC | 0.0436 | 0.0444 | 99.1 | 101 | 100 |
| Cefoperzone | 0.0429 | 0.0489 | 97.4 | 111 | 104 |
| Salvia miltiorrhiza | 0.0166 | 0.0165 | 37.7 | 37.5 | 37.6 |
| Pantoprazole | 0.0594 | 0.0590 | 135 | 134 | 134 |
| Meropenem | 0.0605 | 0.0605 | 137 | 137 | 137 |
| Alprostadil | 0.0591 | 0.0615 | 134 | 140 | 137 |
| Ganciclovir | 0.0548 | 0.0616 | 124 | 140 | 132 |
| Olaparib | 0.0567 | 0.0631 | 129 | 143 | 136 |
| Test Comp. | Peak area of OAT3-mediated ES uptake | | Relative net uptake rate (% of NC) | | |
|  | Sample1 | Sample2 | Sample1 | Sample2 | Mean |
| MOCK-NC | 0.00623 | 0.0106 | NA | NA | NA |
| NC | 0.0496 | 0.0618 | 92.7 | 107 | 100 |
| Cefoperzone | 0.0137 | 0.0191 | 10.8 | 15.8 | 13.3 |
| Salvia miltiorrhiza | 0.0204 | 0.0226 | 24.3 | 28.7 | 26.5 |
| Pantoprazole | 0.0485 | 0.0551 | 80.6 | 93.8 | 87.2 |
| Meropenem | 0.0212 | 0.0219 | 25.9 | 27.3 | 26.6 |
| Alprostadil | 0.0285 | 0.0404 | 53.2 | 51.9 | 52.6 |
| Ganciclovir | 0.0312 | 0.0392 | 45.9 | 55.4 | 50.7 |
| Olaparib | 0.0440 | 0.0426 | 71.6 | 68.8 | 70.2 |
| Test Comp. | Peak area of OATP1B1-mediated E_2_17βG uptake | | Relative net uptake rate (% of NC) | | |
|  | Sample1 | Sample2 | Sample1 | Sample2 | Mean |
| MOCK-NC | 0.000251 | 0.000503 | NA | NA | NA |
| NC | 0.00136 | 0.00200 | 87.0 | 113 | 100 |
| Cefoperzone | 0.00256 | 0.00254 | 169 | 167 | 168 |
| Salvia miltiorrhiza | 0.000366 | 0.000603 | 6.25 | 11.1 | 8.67 |
| Pantoprazole | 0.00192 | 0.00179 | 120 | 110 | 115 |
| Meropenem | 0.00193 | 0.00159 | 97.6 | 94.2 | 95.9 |
| Alprostadil | 0.000686 | 0.000907 | 36.4 | 30.9 | 33.6 |
| Ganciclovir | 0.00279 | 0.00216 | 169 | 155 | 162 |
| Olaparib | 0.00184 | 0.00147 | 114 | 85.0 | 99.3 |
| Test Comp. | Peak area of OATP1B3-mediated E_2_17βG uptake | | Relative net uptake rate (% of NC) | | |
|  | Sample1 | Sample2 | Sample1 | Sample2 | Mean |
| MOCK-NC | 0.000104 | 0.000176 | NA | NA | NA |
| NC | 0.00165 | 0.00205 | 93.5 | 106.5 | 100 |
| Cefoperzone | 0.00114 | 0.00157 | 62.3 | 72.6 | 67.5 |
| Salvia miltiorrhiza | 0.000268 | 0.000349 | 7.57 | 9.09 | 8.33 |
| Pantoprazole | 0.00125 | 0.00145 | 62.1 | 73.2 | 67.7 |
| Meropenem | 0.00126 | 0.00152 | 62.7 | 77.1 | 69.9 |
| Alprostadil | 0.00120 | 0.00121 | 59.3 | 59.9 | 59.6 |
| Ganciclovir | 0.00150 | 0.00187 | 76.0 | 87.9 | 81.9 |
| Olaparib | 0.00118 | 0.00156 | 72.8 | 72.1 | 72.4 |

Note: the peak area of probe substrate transported by MRP2, OAT3, OATP1B1 or OATP1B3 was determined by LC-MS method. Then, the relative uptake rate of probe substrate in the presence of seven drugs was calculated by using equation 7 or 11. When the relative uptake rate was decreased by more than 50%, indicating the potential inhibitory of drug on the transporter.
